# P-1165. Factors Associated with Initial Antifungal Choice for Invasive Candidiasis

**DOI:** 10.1093/ofid/ofae631.1351

**Published:** 2025-01-29

**Authors:** Amira Said, Ankona Banerjee, Ankhi Dutta

**Affiliations:** Baylor College of Medicine, Houston, Texas; Baylor College of Medicine, Houston, Texas; Texas Children's Hospital, The Woodlands, Texas

## Abstract

**Background:**

The Infectious Disease Society of America (IDSA) and European Society of Clinical Microbiology and Infectious Disease (ESCMID) recommend an echinocandin (EC) for first line initial therapy for candidemia. No randomized clinical trial has demonstrated superiority for any antifungal (AF) over another for invasive candidiasis (IC). The EC have become preferred agents due to their favorable side effect profile and early fungicidal activity. Despite current recommendations, there are variabilities in prescribing practices of AF in children with IC. Our goal was to investigate factors influencing initial choices of AF for children with IC.

**Methods:**

We performed retrospective analysis of de-identified data collected as part of the Pediatric Antifungal Comparative Effectiveness Study (PEACE), a collaborative effort by the International Pediatric Fungal Network, conducted 2014 to 2017. 750 patients from 35 centers were included. We looked at demographics, clinical features, risk factors (underlying diagnosis, neutropenia, immunosuppressive medications, stem cell or organ transplant, parenteral nutrition etc) which determine the initial choice of AF within the first 72 hours of IC.

**Results:**

Males comprised 55.6% and White race 58.3%. Majority were non-Hispanic ethnicity (67.7%). Triazoles (TZ) (40.1%) were the most used initial AF followed by EC (33.7%) and amphotericin B (AmB) (17.9%). When EC use was compared to other AF, there was no difference in age or gender. Multivariate logistic regression showed that patients with prior steroid use were more likely to receive AmB than EC. However, patients who had solid tumor malignancy, gastrointestinal insufficiency, neutropenia, prior steroid use and on vasopressors were more likely to receive EC than TZ. Patients who identified as Black were more likely to receive combination AF than an EC alone (OR 2.36, 95% CI 1.03-5.43, *P*=0.016). The most common reason for changing AF from the initial therapy was for step-down therapy followed by treatment failure.

**Conclusion:**

Combination AF use was higher in Black patients. Though overall use of TZ was higher among children with IC, presence of risk factors like steroid use, underlying solid tumor malignancy, neutropenia or vasopressors was associated with EC use. AmB use was not as common as anticipated.

**Disclosures:**

**All Authors**: No reported disclosures

